# Pesticide exposure triggers sex-specific inter- and transgenerational effects conditioned by past sexual selection

**DOI:** 10.1098/rspb.2024.1037

**Published:** 2024-07-17

**Authors:** Veronica Castano-Sanz, Ivan Gomez-Mestre, Eduardo Rodriguez-Exposito, Francisco Garcia-Gonzalez

**Affiliations:** ^1^ Department of Ecology and Evolution, Doñana Biological Station (CSIC), Seville, Spain; ^2^ Centre for Evolutionary Biology, School of Biological Sciences, University of Western Australia, Crawley, Western Australia, Australia

**Keywords:** non-genetic inheritance, rapid environmental change, *Callosobruchus maculatus*, maternal effects, paternal effects

## Abstract

Environmental variation often induces plastic responses in organisms that can trigger changes in subsequent generations through non-genetic inheritance mechanisms. Such transgenerational plasticity thus consists of environmentally induced non-random phenotypic modifications that are transmitted through generations. Transgenerational effects may vary according to the sex of the organism experiencing the environmental perturbation, the sex of their descendants or both, but whether they are affected by past sexual selection is unknown. Here, we use experimental evolution on an insect model system to conduct a first test of the involvement of sexual selection history in shaping transgenerational plasticity in the face of rapid environmental change (exposure to pesticide). We manipulated evolutionary history in terms of the intensity of sexual selection for over 80 generations before exposing individuals to the toxicant. We found that sexual selection history constrained adaptation under rapid environmental change. We also detected inter- and transgenerational effects of pesticide exposure in the form of increased fitness and longevity. These cross-generational influences of toxicants were sex dependent (they affected only male descendants), and intergenerational, but not transgenerational, plasticity was modulated by sexual selection history. Our results highlight the complexity of intra-, inter- and transgenerational influences of past selection and environmental stress on phenotypic expression.

## 1. Introduction

Rapid and novel environmental changes can compromise the viability of natural populations if organisms lack sufficient genetic variation to respond to selection or lack the ability to adaptively shift their phenotype [1–4]. Fortunately, organisms often possess such phenotypic plasticity, increasing their individual survival odds and facilitating the persistence of populations [5–7].

Environmentally induced phenotypic changes are not necessarily restricted to the ontogeny of the organisms directly exposed to an environmental change, but can also be expressed in their offspring across subsequent generations, transmitted through non-genetic inheritance [8,9]. Non-genetic inheritance encompasses heritable information passed across generations that is not directly coded in the primary sequence of DNA [10–12]. Consequently, non-genetic inheritance can be transmitted through a range of parental effects, for instance, related to egg provisioning or parental care, but also through epigenetic mechanisms (typically DNA methylation, histone methylation or non-coding RNAs), which regulate gene expression [13–20]. Non-genetic inheritance contributes to shape heritable phenotypic variation including life-history traits and secondary sexual traits [12,21].

Sexual selection can facilitate or hamper adaptation to changing conditions, depending on the costs of sexual conflict [22–26] and on whether sexual selection is effective at removing deleterious mutations, or reducing the negative consequences of sexual conflict under environmental stress [27–37]. For instance, Parret & Knell [33] showed that strong sexual selection (in male-biased sex ratio populations, as compared with weak sexual selection in female-biased sex ratio populations) provided fecundity and offspring survival benefits that constituted a buffer against increasing temperatures for populations of the moth *Plodia interpunctella*. On the other hand, experimental evolution studies in the seed beetle, *Callosobruchus maculatus,* have shown that the potential benefits of sexual selection are undermined under stressful or changing environmental conditions [37]; or that the effects of sexual selection on population viability can be determined by a complex tension between individual-level adaptation and the genetic benefits of sexual selection at the population level [30].

Research on the influence of sexual selection on adaptation to environmental stress has focused on within- and intergenerational effects. However, it is largely unknown whether intergenerational effects induced by stressful environments contribute to enhancing or hindering adaptation in the face of strong sexual selection [21,30,37,38]. Furthermore, the potential for transgenerational plasticity to modulate the interaction between natural and sexual selection remains to be investigated. Here, we take the first step towards filling that gap.

**Figure 1.**
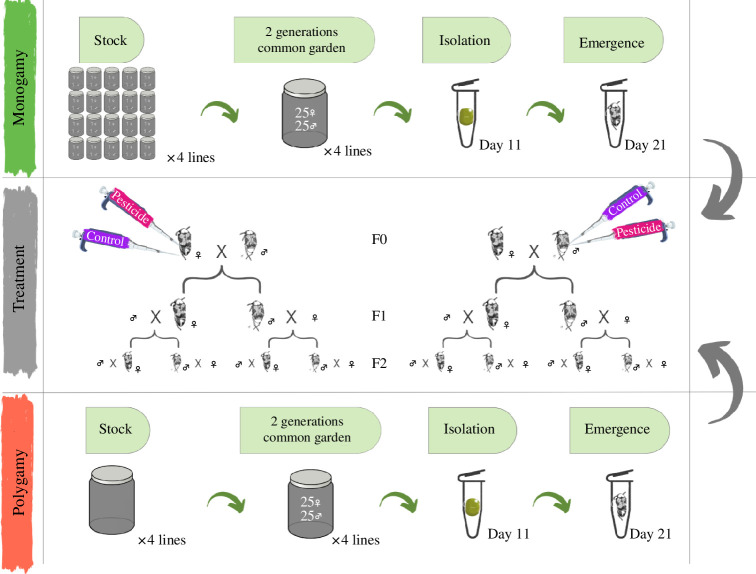
Outline of the 2 × 2 experimental set-up involving selection and exposure to environmental stress treatments. The sexual selection treatment has two levels (polygamous and monogamous evolutionary history) differentiated by the presence or absence of sexual selection for over 84 generations. The pesticide treatment consists of two levels of the toxicant concentration (control and pesticide). Schematics explaining the procedural steps followed from the original populations (polygamy in red at the bottom, monogamy in green at the top) to the application of the toxicant in the F0 generation. Both factors (sexual selection and pesticide) are crossed for the F0 generation. F1 and F2 generations are simply monitored for inter- and transgenerational effects originated in the F0 focal individuals.

Rapid and novel environmental changes such as sudden exposure to toxicants can modify non-genetic inheritance affecting sexual selection and sex differences in life-history traits [21,39], for instance, by disrupting mate choice [40,41]. Specifically, pesticides may trigger hormetic effects and cause phenotypic shifts in subsequent generations through non-genetic inheritance [38,42]. In this study, we carry out a first test of the notion that sexual selection may modulate transgenerational plasticity triggered by exposure to toxic substances. To test this idea, we capitalize on our ongoing work using populations of *C. maculatus* that have experimentally evolved under strong versus relaxed sexual selection and sexual conflict [43]. These *C. maculatus* selection lines were raised under either enforced monogamy or unrestricted polygamy for over 84 generations, therefore, imposing relaxed versus strong sexual selection, respectively [43,44]. In a previous study, we confirmed that non-lethal concentrations of the pesticide deltamethrin trigger transgenerational effects in a population of *C. maculatus* [38]. Here, we exposed males and females from the experimentally evolved lines differing in the strength of sexual selection to the pesticide, hence testing the role of past sexual selection in shaping stress-induced transgenerational effects (figure 1).

We address three main points related to the question of whether sexual selection facilitates or hinders plastic adaptive responses to a stressful environment (exposure to pesticide): (i) we assessed the effects of non-lethal pesticide exposure on life-history traits within the generation exposed (F0); (ii) we addressed whether sexual selection modulates the inter- and transgenerational effects of pesticide on fitness (F1 and F2 generations) and (iii) we tested whether inter- and transgenerational effects are sex specific, i.e. whether they are strongly manifested or transmitted via one sex or the other. Finally, by quantifying the magnitude of transgenerational effects, we provide an assessment of their relative importance in determining adaptation to changing environments.

## 2. Material and methods

The seed beetle *C. maculatus* (Chrysomelidae, Bruchinae) infests dry stored beans and causes damage to grain stores worldwide [45]. Females lay eggs on the surface of dry beans, and upon hatching, the larvae enter the bean, consuming its nutritious part until they reach the chrysalis phase. Once the pupae metamorphose into the adult stage, the beetles open a hole in the bean to exit and then reproduce in a typically polygamous mating system [46–48]. This beetle has a short generation time (3–4 weeks) and high fecundity, with females being able to lay over 80 eggs during their lifetime [46,49]. The conditions under which the beetles are kept in most laboratories, including ours, mimic those to which beetles have been adapting for thousands of generations, infesting stored legumes [35].

We set up and maintained, over the course of several years, an experimental evolution programme in *C. maculatus* in our laboratory at Doñana Biological Station (CSIC, Seville, Spain). As part of our long-term selection experiment, we established a treatment that imposed variation in the opportunity and intensity of sexual selection by enforcing monogamy (thereby relaxing sexual selection and sexual conflict) in four replicated populations, while keeping polygamy (thereby maintaining intense sexual selection and sexual conflict) in another four selection lines, for over 80 generations. In the polygamous lines, sexual interactions and matings occur in an unrestricted way among all the individuals and thus, intense pre- and postcopulatory sexual selection operates in these populations. In the monogamous lines, beetles are forced to mate on a one-to-one basis; sexual selection and ensuing sexual conflict are thus minimized because in these lines, there is no room for pre- or postcopulatory female choice, or for male–male competition. Population size for each of the selection lines has been kept constant to avoid undesired effects arising from genetic drift (50 founding individuals, 25 females and 25 males, for every population and generation). Full details of the experimental evolution protocol and its expected impact on the selective forces (intensity of sexual selection, softness of selection and scope for fecundity selection) acting on the replicated populations from the different selection regimes are provided in Rodriguez-Exposito & Garcia-Gonzalez [43] and Canal *et al*. [44].

This set-up gave us the opportunity to test if divergent regimes of sexual selection (after 84 generations of continued selection) modulate transgenerational plasticity in response to environmental stress (pesticide exposure). The experimental design hence consisted of a 2 × 2 design in which we crossed the two selection regimes (monogamous, henceforth Mono, or polygamous, henceforth Poly) with exposure/lack thereof of a non-lethal concentration of pesticide (figure 1).

Before beetles were subjected to the environmental stress treatment, the different selection lines underwent two generations of common garden breeding, to minimize the probability that any potential parental effects associated with a particular kind of mating or breeding obscured the assessment of the effects of selection, i.e. to increase our confidence that any transgenerational responses could be attributed to genetic assimilation [50,51]. Common garden breeding was carried out, first by duplicating the number of inoculated beans collected from each line to give rise to the next generation (i.e. 300 beans from each of the eight populations; see Rodriguez-Exposito & Garcia-Gonzalez [43]) and then by establishing a spare set of eight populations that were all bred under polygamous conditions for two generations, while maintaining the selection experiment with the original populations. After those two common garden generations, and to obtain focal virgin individuals of known age, we isolated three times as many infested beans as beetles were needed in our pesticide experiment. The isolation procedure was identical to that employed by the selection experiment and consisted of extracting inoculated beans with only one egg on them from the containers 11 days after oviposition, and reallocating them individually in perforated Eppendorf tubes to ensure the virginity of the adults. This isolation protocol also allowed us to know the exact age of the focal adult beetles, since adult emergences from the bean were checked daily.

Five virgin males and five virgin females were randomly selected within each of the eight selection lines that underwent common garden breeding as the focal individuals to be allocated to each of the pesticide exposure levels (exposed and control) in our experiment to non-lethal concentrations of the pesticide deltamethrin (COMBO Deltamethrin 2.5% w/v, Sarabia). Pesticide application was restricted to these beetles (the parental generation, F0), but subsequent effects were tracked over three generations (F0, F1, F2). Thus, 160 virgin beetles of *C. maculatus* (80 females and 80 males), with different evolutionary histories regarding sexual selection regimes (Mono, Poly), were exposed to different levels of previously tested non-lethal concentrations of the pesticide [43], inside a flow cabinet for 24 h, at 23 ± 1°C, 12:12 Light : Dark and 35% RH. Two different pesticide treatments were given: (i) control treatment (C), without pesticide, exposed to distilled water; (ii) pesticide treatment, 2 g/l deltamethrin in aqueous solution (P). In short, the exposure was done randomly assigning five individuals from each sex from each of the eight lines (four Mono and four Poly) to each treatment, having a total sample size of 160 parental exposed beetles in our experiment. Individuals at the moment of pesticide/control exposure were between 1 and 4 days old.

For the pesticide exposure, the ends of cotton swabs were impregnated with 30 μl of the pesticide or water and put individually into 2 ml opened Eppendorf tubes, which would be filled with the evaporation of the pesticide solution shortly after closing the tubes and throughout most of the 24 h of exposure. Next, beetles were introduced to a second perforated Eppendorf tube with the bottom removed and stacked onto the first tube, with a mesh barrier between them, keeping the cotton swab in the lower compartment and avoiding direct contact of the beetles with the pesticide. After the exposure phase, beetles were individually allocated to 26 ml perforated plastic containers with ad libitum beans (around 60 beans per vial) to be used as oviposition substrate. After the exposure phase, and until death, beetles were kept in walk-in climate chambers (Fitoclima 10 000 EHF, Aralab) at a constant 29°C temperature with 40% humidity and a 12:12 Light : Dark cycle, i.e. the conditions at which all animals from the experimental evolution programme had been cultured since the start of the selection experiment. Each individual shared the recipient with a non-exposed tester mate (male or female), all sourced from a standardized heterozygote tester line that was generated by crossing two near-isogenic lines that had been generated following 15 generations of full-sib mating (all individuals used in the generation of the near-isogenic lines were drawn from the stock population, i.e. they were individuals from outside the selection experiment). The use of these genetically homogeneous tester individuals minimizes sampling variance and ensures that the potential transgenerational effects are not obscured by genetic or non-genetic variation in the individuals that are used as mates [52–54]. Mating and oviposition were allowed for 48 h. Afterwards, focal males were individually reallocated to Eppendorf tubes to track their longevity, whereas mated females (focal females that were mated to tester males, or tester females who were mated to focal males) were kept in their container to determine longevity, fecundity and lifetime reproductive success (LRS) for each of them.

We recorded life-history traits (longevity, fecundity and LRS) on the generation exposed to pesticide treatment (F0), and on their offspring (F1) and grand-offspring (F2). The F1 and F2 generations were not exposed to pesticide, but conditions for isolation, housing and mating were similar to the parental generation. The sample size doubled in each generation because we selected both a son and a daughter from each cross in each generation (160 focal beetles in F0, 320 focal beetles in F1 and 640 focal beetles in F2), hence allowing us to test for differences between the sexes, in the F0, F1 and F2 generations.

### (a) Variables of interest

We recorded the longevity of all beetles by individually monitoring survival on a daily basis from adult emergence until their death. We estimated total fecundity by carefully checking the presence of eggshells on each bean within every vial, on day 11 after oviposition. Fecundity was measured for the F0 generation only as our focus was primarily on the total number of adult offspring produced along life (LRS), and fecundity measures could not be attained for F1 and F2 generations owing to the large sample sizes for individuals assessed in those generations. LRS could be measured as viable eggs hatched in a 28 day interval [43,55]. As mating and oviposition times were controlled in our experiment, containers were frozen 28 days after the death of the female (the last possible day for oviposition). For those females who lived longer than 14 days, containers were always frozen before the predicted emergence date of the next generation (corresponding to day 42 from the first oviposition date). LRS was measured for all beetles in all generations by accounting for the lifetime number of adults produced by each female (either focal females or tester females mated to focal males).

Dry body weight was measured after death for all individuals, focal or tester, and in all generations, with a Sartorius Cubis MSA 6.6S microbalance (accuracy 0.001 mg, Sartorius, Goettingen, Germany). Beetles were frozen at −20°C when found dead, for a period of several weeks until the end of the experiments. The animals were then thawed and dried for 1 week at 40°C (so as to remove variation in weight owing to time elapsed between death and freezing) before being weighed.

### (b) Statistical analyses

Our response variables were longevity, fecundity and LRS. Since the variables are count variables (days alive, number of offspring), we fitted generalized linear mixed models (GLMMs) with a Poisson error distribution, using the *glmer* function in the package *lme4* v. 1.1.35.1 [56]. However, in addition to problems of overdispersion, the validation of Poisson models was poor. For this reason, we ran linear mixed models (LMMs) using the ‘lmer’ function, which consistently performed better in terms of goodness-of-fit. The validation of LMMs was satisfactory in tests using simulated residuals (see the last paragraph in this section), and results were similar regardless of whether LMMs or Poisson GLMMs were run. Therefore, we present the results from LMMs. LMMs were run on untransformed or transformed (Box-Cox or cubic root transformation) response variables, depending on what produced the best model validation (see the last paragraph in this section, and the electronic supplementary material, tables S1-S6). Models included pesticide (Control, 2 g/l) and selection regime (monogamy, polygamy) as fixed factors, and selection line ID as a random factor (eight lines, four for each of the two selection regimes). Given the association between body size and fecundity, and to account for survival–reproduction relationships [43,49,57–60], we included longevity as a covariate in models testing effects on fecundity and LRS, body weight of the focal individual as a covariate for longevity of those focal individuals, female fecundity as a covariate in F0 longevity models (fecundity was only measured in the F0 generation) when the focal individual analysed was a female, and female body weight (focal or tester) as a covariate for fecundity and LRS models. The age at mating (1–4 days old) of the individual tested was also included in the models. We applied mean-centring of numerical covariates in the models. Individuals with incomplete information regarding response and predictor variables were excluded from the analyses (final sample sizes for each model can be found in the electronic supplementary material, tables S1-S6). All covariates were included as control predictors, and so random slopes were not modelled, as interactions between these control predictors and the treatments were not present in the model (they were not the focus of the analyses), and this procedure is expected not to inflate type I errors [61]. To ease the interpretation of results and also because in this species males and females have distinct life histories [44,62], we conducted the analyses separately for each sex of the focal individual, and by the sex of the parent/grandparent (for F1 and F2, respectively) initially exposed to treatment.

There were a few cases of LRS equal to zero (4.62% of data out of a total of 887 LRS data points across the three generations). Such low incidence of infertile matings can be mostly attributed to infertility or sickness, rather than to treatment effects (see [43] for similar rates of unproductive matings in the species and [63] for infertility rates across taxa). Thus, we eliminated LRS = 0 data from the analyses. We, nonetheless, checked that the inclusion or exclusion of these data did not qualitatively affect the results. There was a loss of 30% of the longevity data in the F2 generation owing to a malfunction in the data synchronization system of our cloud storage, but we still attained a sample size of 439 data points for grand-offspring longevity. We confirmed that data loss was random with respect to the distribution of longevity and restricted the analyses to individualswith complete information regarding response and predictor variables (*n* = 407 grand-offspring). The statistical significance of fixed effects was calculated using Type II (Type III if significant interactions) Wald chi-square tests.

Model diagnostics were carried out by calculating (using a simulation approach) and plotting scaled (quantile) residuals using the package ‘DHARMa’ [64]. When differences were detected, we estimated effect sizes (Cohen’s *d*) from marginal means from the model and their associated 95% CIs with bootstrapping, using the package *emmeans* v. 1.8.8 [65], to quantify the magnitude of differences in the response variables among groups. We controlled the false discovery rate of tests conducted on the F1 and F2 data using the *p.adjust* function in R using the Benjamini–Hochberg procedure [66]. We corrected *p* values within each generation and sex exposed to pesticide treatment. In the models, the reference level for the pesticide treatment was always the control level, and the reference for the sexual selection regime was polygamy as this is the natural mating system of *C. maculatus*. All analyses were run in R v. 4.2.0 [67].

## 3. Results

We first present results on the effects of sexual selection regime and pesticide exposure on the parental generation (within-generation or intragenerational effects). We subsequently present results on the consequences of sexual selection regime and pesticide exposure of the parental generation on their offspring’s fitness (intergenerational effects), and grand-offspring’s fitness (transgenerational effects).

### (a) Within-generation effects

**Figure 2.**
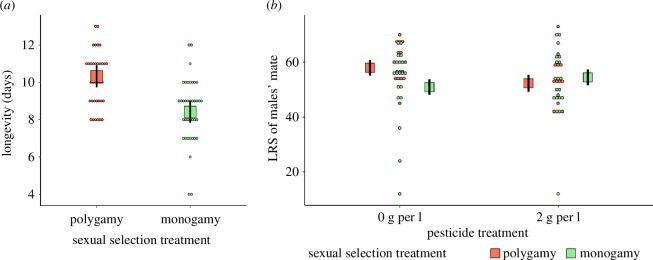
Results from F0 generation. (*a*) Longevity (days) of males. (*b*) LRS (number of adult offspring produced) of focal males’ mates. Marginal means (boxes) and associated s.e. (black lines) are shown.

Male and female longevities were not affected by pesticide exposure or the interaction between pesticide treatment and sexual selection regime. However, regardless of pesticide exposure, males from polygamous populations lived significantly longer (22.7%) than their monogamous counterparts (*X^2^* = 5.07, d.f. = 1, *p* = 0.024, electronic supplementary material, table S1, figure 2*a*, Cohen’s *d* (95% CI) = 1.41 (−0.144, 2.95)). Furthermore, fecundity and LRS models showed that selective regime or pesticide exposure had no effect on neither females nor males’ mate fecundity and that LRS of females was not affected by experimental factors either. However, the interaction between pesticide treatment and sexual selection regime on the LRS of the males' mates was marginally non-significant (*X^2^* = 3.72, d.f. = 1, *p* = 0.054, electronic supplementary material, table S2). In the absence of pesticide, the LRS of the males’ mates was 14% higher when males were sourced from polygamous populations than when they came from monogamous populations. However, the reverse was true under pesticide exposure, with the LRS of the males’ mates being 4.2% higher for males from monogamous populations compared with their polygamous counterparts (figure 2*b*). This interaction reflects that sexual selection may be beneficial in adapted populations under stable environmental conditions, but it may be costly when populations are exposed to stressful environments.

### (b) Intergenerational plasticity

**Figure 3.**
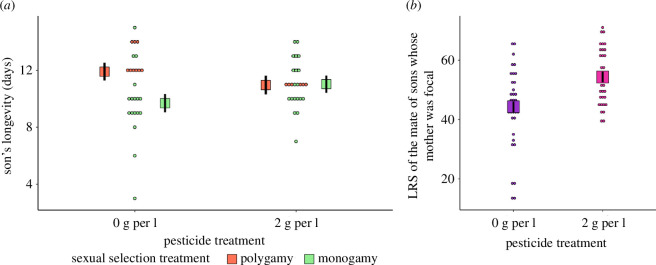
Results from F1 generation. (*a*) Longevity (days) of the sons generated by focal males. (*b*) LRS (number of adult offspring produced) of sons’ mates whose mothers were focal. Marginal means (boxes) and associated s.e. (black lines) are shown.

The longevity of the sons generated by focal males was affected by an interaction between the sexual selection regime and pesticide exposure (*X^2^* = 6.28, d.f. = 1, *p* = 0.044, electronic supplementary material, table S3, figure 3*a*). Longevity was 22.9% higher for sons from polygamous populations than for sons from monogamous populations when their fathers were not exposed to the pesticide (Cohen’s *d* (95% CI) = 1.19 (0.10, 2.28)). However, when the fathers of F1 males were exposed to the pesticide, the difference in sons' longevity between the two levels of the sexual selection treatment was negligible (sons from exposed fathers from monogamous populations lived 0.6% longer than sons from exposed fathers from polygamous populations). This interaction again suggests good gene benefits of sexual selection that may be nullified under environmental stress. The longevity of offspring from exposed mothers and the longevity of daughters from exposed males was not affected by the factors (electronic supplementary material, table S3).

There were also intergenerational effects impacting LRS. Specifically, when the mothers of F1 males were exposed to 2 g/l of pesticide, their sons’ mates had a 22.6% greater LRS than the corresponding counterparts generated by control mothers (*X^2^* = 14.80, d.f. = 1, *p* = 0.001, electronic supplementary material, table S4, figure 3*b*, Cohen’s *d* (95% CI) = 0.93 (0.4, 1.46)). This result reveals a counterintuitive positive intergenerational effect triggered by pesticide exposure. As for sexual selection effects on LRS, daughters from fathers evolving under polygamy had higher LRS than daughters from fathers from monogamous populations, but this result did not stand false discovery rate correction (electronic supplementary material, table S4).

### (c) Transgenerational plasticity

**Figure 4.**
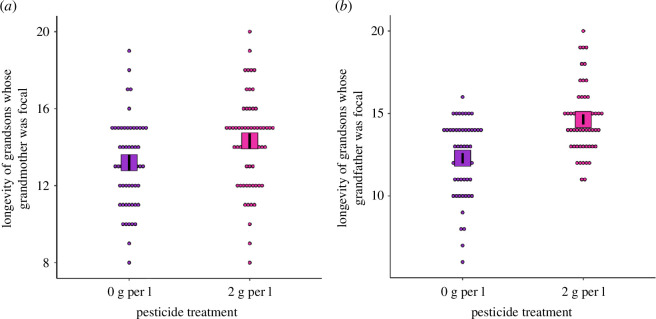
Results from F2 generation. (*a*) Longevity (days) of grandsons whose grandmothers were either exposed to pesticide or water. (*b*) Longevity (days) of grandsons whose grandfathers were either exposed to pesticide or water. Marginal means (boxes) and associated s.e. (black lines) are shown.

The longevity of grandsons was significantly affected by the exposure to pesticide of their grandparents, regardless of the sex of the focal grandparent (electronic supplementary material, table S5), revealing transgenerational influences of toxicants. Specifically, the longevity of grandsons whose grandmother was exposed to 2 g/l of deltamethrin was extended on average by 8.3% (*X^2^* = 8.61, d.f. = 1, *p* = 0.017, electronic supplementary material, table S5, figure 4*a*, Cohen’s *d* (95% CI) = 0.51 (0.08, 0.95)); whereas the longevity of grandsons whose grandfather was exposed was increased by 18.7% (*X^2^* = 38.75, d.f. = 1, *p* < 0.001, electronic supplementary material, table S5, figure 4*b*, Cohen’s *d* (95% CI) = 1.22 (0.76, 1.70)), compared with their control counterparts. Longevity was not affected by sexual selection regime or its interaction with pesticide exposure. Grand-offspring’s LRS was not affected by any factor, although there was an interaction between selection regime and pesticide exposure affecting the LRS of grandsons' mates, which did not stand the correction for falsediscovery rate (electronic supplementary material, table S6).

## 4. Discussion

Whether sexual selection facilitates adaptation and population persistence in the face of sudden environmental change remains widely debated [27–29,32,34,37,68–71]. We argue that to answer this question, it is key to consider the potential transgenerational effects that environmental disturbances may trigger. Using experimental evolution, we conduct a first test of the notion that the strength of sexual selection may modulate transgenerational plasticity elicited by stressful environments. Our results showed that an environmental disturbance, in the form of non-lethal exposure to a commonly used pesticide, elicits not only inter- but also transgenerational effects on longevity and fitness, although to different extents in the different generations. We also found that intergenerational effects on longevity were modulated by evolutionary history in terms of sexual selection and that the mating system itself, on top of shaping longevity in a sex-specific way, can induce intergenerational effects on fitness.

### (a) Intragenerational effects

We found that 84 generations of divergent sexual selection had no direct noticeable effects on female fitness (longevity, fecundity or LRS) when female fitness is assessed independently of male-driven effects. However, our results show a divergence in the ability of males from the two selection regimes to induce higher or lower fitness on females depending on environmental conditions. Under benign conditions (i.e. no pesticide exposure), males from polygamous populations conveyed higher fitness (LRS) to their mates than their monogamous counterparts, but this was not the case under toxicant exposure. These results are in line with the general expectation that strong sexual selection may improve female fitness through the benefits of female mate choice, potentially leading to population viability increases through good gene effects and the condition dependence of male sexual traits [29–31,70,72–75].

Strong sexual conflict and intense sexually antagonistic selection are common in *C. maculatus* [43,76–79], and consequently, females interacting and mating with males from a polygamous lineage may experience costs resulting in an overall reduction in net fitness [80,81]. Previous research using the same selection lines found that tester females from outside the selection experiment exposed to lifelong male harassment and mating attempts from several males from the polygamous selection lines pay higher costs (i.e. they have lower LRS) than tester females from outside the selection experiment exposed to males from the monogamous populations [43]. Such reproductive loads are a signature of sexual conflict [82,83]. Here, we exposed females to much less harmful conditions (one female–one male cohabitation during 48 h), and this scenario revealed the benefits of sexual selection, at least under benign conditions. However, the pattern was reversed after exposure to pesticide. In such stressful conditions, the LRS of females mated to males from monogamous lines was higher than that of females mated to males from polygamous populations. This interaction suggests that sexual selection may be a hindrance for adaptation to changing environments. In systems with strong sexual antagonism, it is presumed that the detrimental consequences of sexual conflict could be ameliorated under stress because in harsh environments, male and female interests are expected to converge, especially when considering that body condition may underlie the expression of male harm [35,36,83,84]. Recently, we found that sexual selection can buffer the negative influence of population subdivision on adaptation to warm temperatures [85]. The present study, however, suggests that sexual selection may be ineffective in protecting populations that face rapid environmental change. These results are in line with previous findings in *C. maculatus* and other animals showing that sexual selection and its associated benefits may be disrupted in maladapted populations [30,37]. The results in other systems also suggest that the benefits of sexual selection to population fitness may not be large when the environment changes rapidly [33].

Lastly, while testing for intragenerational effects of sexual selection was not the aim of the present work, our tests showed that intense sexual selection and conflict select for longer lifespan in males but not females. Our study thus contributes valuable data to address the issue of whether sexual selection and conflict influence the evolution of lifespan and ageing—an open question that necessitates empirical evidence [86–90]. Interestingly, this test provides a magnitude for a direct effect of selection on longevity that can be related to the magnitude of the inter- and transgenerational effects on longevity observed in the other sections of our study (see §4d).

### (b) Intergenerational effects

We observed a signal for intergenerational effects of sexual selection. Specifically, daughters from fathers evolving under strong sexual selection (i.e. under polygamy) had higher LRS than daughters from fathers from monogamous populations (electronic supplementary material, table S4). However, this result does not stand after correction for false discovery rate and so it needs to be interpreted with caution. It may be argued that our correction may be overly conservative and therefore, we discuss some of its implications. If the effect is true, this would be consistent with classical good gene (indirect) benefits of female choice and female multiple mating, to the extent that the increased LRS of daughters can be the result of increased viability or overall quality of daughters sired by males from polygamous populations. Such intergenerational effects could also be the outcome of non-genetic inheritance [10,11,91], because of ejaculate-mediated paternal effects enhancing daughter’s fitness or the egg-to-adult viability of the daughters’ offspring [20,53,92–95]. For instance, previous research in *Drosophila melanogaster* has shown an increase in the productivity of daughters but not the reproductive success of sons, when mothers increase the number of interactions with non-sires, implying transgenerational effects from increased female sexual interactions that impact the productivity of future generations [53]. Our study was not designed to partition the variance underlying the observed intergenerational effects into components of genetic and non-genetic inheritance, but this would be a fruitful avenue for future research via the implementation of paternal and maternal half-sib designs paired with functional epigenomic analyses.

Sexual selection regime and pesticide exposure interacted in the way they affected the longevity of the sons generated by focal males. Longevity was higher for sons from polygamous populations than for sons from monogamous populations when their fathers were not exposed to the pesticide, but lifespan was similar for sons whose fathers had been exposed to the toxicant, regardless of their sexual selection evolutionary history. This result, again, is suggestive of good gene benefits of sexual selection that may be nullified under environmental stress.

Pesticide exposure resulted in intergenerational effects such that LRS of sons’ mates increased when their mothers were exposed to pesticide. Pesticide exposure triggers detoxification processes in beetles that may result in resource allocation conflicts, which can themselves bear consequences for subsequent generations [96–98]. Furthermore, it has been shown that some chemicals, like pesticides, can have hormetic effects triggering responses contrary to expectation when applied at sublethal doses [38,99–102]. The application of such toxicants may therefore have unforeseen consequences in subsequent generations and even result, counterintuitively, in population increases [38,42].

### (c) Transgenerational effects

Our results show that there are important and far-reaching transgenerational effects of non-lethal pesticide exposure, as we detected an increase in the longevity of grandsons whose grandparent was exposed, regardless of their sex. These results are consistent with Castano-Sanz *et al*. [38], who showed that grand-offspring lived longer after parental exposure to deltamethrin. Extended longevity implies higher chances for additional matings, and consequently an increased number of descendants. This is especially so in males, owing to their high potential reproductive rates and their lower investment in gametes and the absence of parental investment in the species. In addition to the potential for an increased number of matings associated with increased longevity, there could also be a small increase in the LRS of females mated to grandsons whose grandparents were exposed to pesticide. The repercussions of these findings for pest control and agroecosystem management are far-reaching, as they reveal that the use of toxic substances to control pest populations may backfire because of unanticipated transgenerational effects that can actually imply an increase in population fitness [38,96]. More broadly, our results support the view that new approaches considering generational toxicity are key not only to understand the transgenerational inheritance of disease [103] but also population dynamics.

Interestingly, while we found signals for intergenerational effects associated with evolutionary history in terms of sexual selection intensity, we failed to detect any transgenerational effects linked to sexual selection. The absence of transgenerational effects from sexual selection can be due to several reasons. First, they may be non-existent or small and undetectable with our experimental design and sample sizes. Second, they may have gradually faded away (i.e. large effects from F0 to F1, and decreasing in magnitude from F0 to F2). Third, as in our experimental design we enforced random monogamous matings to all experimental individuals (hence, somehow halting the action of sexual selection), we can consider that the enforcement of monogamous matings to individuals from polygamously evolved lines constitutes itself an environmental change (in terms of socio-sexual interactions), which may have nullified transgenerational effects associated with strong sexual selection.

### (d) The magnitude of inter- and transgenerational effects is comparable to the direct effects of selection

Regardless of the mechanisms underlying the effects that we have uncovered, it is interesting to note that the magnitude of the transgenerational effects induced by non-lethal exposure to pesticide on longevity (up to 18.7%, Cohen’s *d* = 1.22 (0.76, 1.70)), is comparable to the magnitude of the intergenerational effects of sexual selection on longevity in benign environments (22.9%, Cohen’s *d* = 1.19 (0.10, 2.28)), and notably, to the magnitude of the direct effects on longevity derived from sexual selection over >80 generations (22.7%, Cohen’s *d* = 1.41 (−0.144, 2.95)). This is remarkable because it shows that rapidly triggered stress-induced inter- and transgenerational effects on traits may not be mere nuanced modifications of traits, but actually be comparable to the effect of selection over a reasonably long period of time.

### (e) Sex specificity of transgenerational effects

Transgenerational plasticity may originate due to independent maternal or paternal environmental influences, and the responses in subsequent generations may also be sex specific [9,38,98,104,105]. Currently, there is increasing interest in understanding the extent and implications of such sex specificity. For instance, using individual-based models, Burke *et al*. [39] emphasize that complex patterns of adaptive and non-adaptive sex-specific transgenerational effects can arise depending on the existence or absence of sexually antagonistic selection. Maternal and paternal effects can have different effects on offspring, and sons and daughters may also respond differently to environmental cues. For instance, mothers may differentially allocate the amount of hormones present in eggs [106,107]. However, while research on maternal effects has been extensive [15,108,109], paternal effects have been comparatively less studied, and until recently, they were largely ignored [17,20]. Indeed, the notion that males could influence offspring fitness beyond DNA sequence transmission in species without paternal care was considered an impossibility until very recently. Now, it has been clearly established that fathers may transmit environmental information through ejaculate-mediated paternal effects [20,110,111], including the non-sperm fraction of the ejaculate [18,94,95]. Our study was designed to compare transgenerational plasticity inherited either maternally or paternally, as well as to assess its effects on either male or female offspring and grand-offspring. In the F0 generation, we found a sex-specific response concerning the sexual selection evolutionary regimes and their interaction with the pesticide, as only males were affected. In the F1 generation, the pesticide treatment only had intergenerational effects on sons, whereas the sexual selection regime only had an impact on daughters, and just via fathers, not mothers. Finally, the pesticide had a transgenerational impact on grandsons regardless of whether the exposed grandparent was male or female. These inter- and transgenerational effects were also trait specific, as different traits were affected across different generations. Future research on the mechanisms underlying this sex specificity, and varying trait sensitivity, is warranted.

In conclusion, empirical investigations of the role of sexual selection and sexual conflict underlying transgenerational plasticity are lacking. Here, we conducted a first test, using experimental evolution, for the involvement of sexual selection in promoting or triggering transgenerational plasticity in the face of rapid and stressful environmental change. Our results highlight the complexity (including sex specificity) underlying within-, inter- and transgenerational influences of sexual selection and environmental stress.

## Data Availability

Data and scripts to analyse the data are available at Dryad and Zenodo [112]. Supplementary material is available online [113].
